# Beyond Risk Considerations: Where and How Can a Debate About Non-safety Related Issues of Genome Editing in Agriculture Take Place?

**DOI:** 10.3389/fpls.2018.01724

**Published:** 2018-11-26

**Authors:** Sarah Bechtold

**Affiliations:** Institute Technology-Theology-Natural Sciences (TTN), Ludwig-Maximilians-University Munich, München, Germany

**Keywords:** ethics, genome editing, food labels, new breeding techniques (NBTs), free choice, public debate, value decisions

## Introduction

Having the potential to realize breeding objectives that were out of reach so far, genome editing (GE) surely constitutes a major advancement in the field of plant research, especially for the agricultural sector. Only recently has the debate about GE and its possible use in food and feed production transcended the scientific circle toward a political discussion. Considering the discussions about genetically modified organisms (GMOs) in the past, it is very likely that the public debate about genome edited food and feed products will be highly controversial. This article will show that the debate about genome editing is already risk-focused and that the resulting confinement structurally hampers a sound discussion of the values that are at stake. In contrast, to this development I argue that a comprehensive deliberation of values is needed in the context of genome editing in agriculture. Moreover, those deliberations should be separated from risk analysis and allow for individual decisions within our value system. Finally, I will discuss food labeling and consumer choice as an institution to support communication about values and to broaden the perspective on the agricultural use of genome editing and its products.

## The debate about genome editing is risk focused—contentwise and structural

Every human action, but especially actions with a wide range of effects that have not yet been tested, such as the use of new genome editing technologies, are inevitably linked to uncertainty and ignorance. Therefore, it should not come as a surprise that risk issues are prominent in the debate about genome editing. However, they are addressed very differently in various settings of the discussion. These differences can partly be traced back to different notions of risks. Within the scientific discussion, the risk of an action is defined as the product of the extent of damage and the probability of its occurrence (Knight, 1921). This notion leads to a gradual risk concept that allows for empirical assessment and the balancing of risks and opportunities for action. However, it tends to cover only those risks that are accessible from the scientific perspective (Jasanoff et al., 2015). Accordingly, scientific investigation has a focus on measures to reduce foreseeable risks arising, for example, from off-target effects of the technique (Kadam et al., 2018). In contrast, within the public debate the term risk is used and understood in a much broader sense, to include unknown and unforeseeable risks. Moreover, the very same problems of the technology, such as off-target effects, are perceived as a black box that scientists are principally unable to penetrate and therefore become principally unpredictable hazards (Testbiotech Background, 2018). For this wide notion of risk the balancing approach of risk management, favored by scientists, is not applicable. Instead many public actors promote a strong precautionary strategy^1^ to avoid, in extreme cases, any possible risk regardless of possible benefits.

This wide, colloquial notion of risks as hazards is highly problematic, but very efficient in terms of opinion formation. Thought through to the end this notion—in combination with the strong interpretation of the precautionary principle—leads toward the acceptance of any existing grievance, because unknown and unforeseeable risks of actions to improve bad situations could always exceed the existing problem. By raising apprehensions and fears, especially when human health is at stake, safety-related arguments become very compelling and are often used as discussion-terminating arguments without further need of proof. However, besides human health, other goods, such as ecological issues, autonomy, and matters of social justice are also prominent in the public debate about genome editing. The extent to which those issues are relevant for the appraisal of the technology and the way in which they are addressed not only depend on the technology itself, but also on the historical and socio-cultural setting of the debate in question (Torgersen, 2009; Sassatelli and Scott, 2010). In the case of genome editing, the initial scientific framing of the GMO debate (Jasanoff et al., 2015) prompted a risk perspective on the whole spectrum of issues and arguments concerning genome editing in agriculture. This means that the focus lies on the difference between a status quo, which is postulated a neutral point of reference, and potential deterioration of the status quo due to the technological innovation. The predominance of the risk perspective has several negative impacts: First of all, it narrows the scope of the discussions toward safety-related issues. Second, questions of personal preferences and lifestyle choices are marginalized. Thirdly, the value-nature of the goods at stake–the wellbeing of humans, human societies and ecological societies–and the conflicts that can emerge between those goods fall out of focus within the prevalent discussion structure. These effects strongly suggest an improvement of the debate by preventing that risks are perceived as hazards without further ado. At the institute Technic-Theology-Nature science (TTN) we investigate how ethics can contribute to that improvement^2^. While Jasanoff et al. endorse to open up the risk debate for societal apprehensions in order to overcome the constraints of a purely scientific perspective (Jasanoff et al., 2015), we argue in favor of a risk-independent value discussion.

## Why do we need a risk-independent debate about values in the context of genome editing in AGRICULTURE?

Many voices–involving proponents and critics of agricultural application of genome editing–already claim that value considerations should be acknowledged in the admission process of genome edited products for various reasons (Myhr and Myskja, 2018; Röcklingsberg and Gjerris, 2018). For one, scientists working with genome editing techniques often argue that those techniques could help us to realize higher-level values, such as human health, protection of the environment or sustainable agriculture, which were not achievable by other breeding methods at all or within a certain time frame. In other words: social-political goals may be achieved by cultivating genome edited plants and livestock. Genome edited plants, like a mildew-resistant wheat, could, for example, contribute to reducing the use of pesticides. However, many breeding goals are relevant only to specific societal groups, like allergen-free peanuts, or can even hinder the achievement of societal goals, if they for instance promote herbicide resistance thereby increasing the use of those chemicals. In addition, not only the nature of genome edited products, but also the practices and circumstances of their cultivation and distribution will shape the impact of the new technologies. Therefore, also these aspects should be questioned for possible threats to social values, such as justice, autonomy and respectful interaction with nature. Finally, giving full consideration to ethical, social and sustainability related aspects of genome editing is crucial for the acceptability of the technology. Moreover, neglecting ethical, social and sustainability related aspects could be interpreted as a political failure, especially by people approving a reasonable employment of the new technologies.

For these reasons, value-based arguments should not be discredited as mere expressions of irrational attitudes (Pirscher and Theesfeld, 2018). However, the discussion of value arguments requires a different procedure and different solution strategies than a scientific risk discussion. Disagreement about scientific knowledge is at least theoretically easy to overcome because findings become wrong and irrelevant when contradictory evidence has proven to be right. In contrast, discord about values is much more durable and leads to continuing conflicts because conflicting values can exist side by side. Moreover, they only become significant within the context of a value system including other, potentially conflicting values. Although values and their fundamental relations are commonly shared within a society and thereby have normative potential, individual members of a society frequently differ in their point of view when it comes to indissoluble value conflicts. Because value decisions can differ within the scope of ethically acceptable choices, consensus solutions cannot be considered as the ultimate objective for societal value conflicts (Bogner, 2015). This particularly holds true, if the conflict at stake touches on the lifestyle of individual persons including their food choices. Instead societies need tolerance to enable people with different attitudes, interests and preferences to live together. This is an important difference between the appropriate handling of safety issues and value conflicts about genome editing in agriculture: If someone, by her choice of production or consumption, exposes people or the environment to unreasonable risks, I will legitimately reject that behavior. In contrast, if someone takes a decision in accordance with values that do not parallel my own, or opts to pursue goals that we may share through different means, it remains necessary that I defer to her specific value orientations to a certain extent. Tolerance here means that I attempt to understand and respect how and why her thought processes do and need not mirror my own. Hence, societal value debates do not come to a single result that defines the universally applicable action, but should allow for multiple options that can persist in parallel. But how and where can that plurality be implemented?

## Food labeling as an institution for value deliberations concerning genome edited products

The proposal of integrating value considerations into the admission process of genome editing and its agricultural products will face a number of problems. A classical objection is that value criteria are vague and subjective (Zetterberg and Björnberg, 2017) and therefore not easy to justify. Value-sensitive regulations must also be defended against the reproach of nudging the public in a paternalistic way when they are state-imposed. With respect to the claim of providing a plurality of value attitudes within a society, one of the greatest drawbacks of implementing value considerations in the regulation of the production of genome edited foods is that it would reduce the spectrum of available products and thereby impose a concrete constraint on consumer decisions. This procedure not only elides how and why positive freedom, i.e., the prerequisite of having substantial options, is ultimately crucial for instituting freedom of choice (Taylor, 1979), but is particularly problematic in relation to a concept of social freedom (Honneth, 2014) which is claimed fundamental to communities based on liberality and solidarity and implies that decisions–especially governmental decisions–should be judged by the extent to which they foster the freedom of others. These restraints of freedom can be sufficiently justified for immediate safety reasons regarding human health or the environment. However, it is not in accordance with value decisions, derived from prioritizing one value over another, yet creditable value. While closing down the debate is necessary in the first case, opening up would be adequate in the latter (Stirling, 2008). One way to open up the debate about food and its production not mentioned by Stirling is to allow for value decisions on the level of consumption. For example, by labeling genome edited products and/or foods produced without that technology in a way that allows for communication about associated values. Furthermore, in the light of rapid technological development time pressure constitutes a serious problem for comprehensive and well-considered regulatory decisions regarding the agricultural use of genome editing. Labeling, instead, could stagger the processes of deliberation allowing for cautious governance of the new breeding technologies. In other words, allowing for case-by-case decisions in the supermarket could lessen the freedom-reducing effect of national governance decisions and render them adaptable to development in the public attitude, because also consumers who did or could not engage in the public debate are continuously able to make or change their decisions. However, labeling and consumer decision does not render scientific expertise and governmental institutions unnecessary. As with every new technology, genome editing for agriculture requires safety precautions concerning human health, society and ecosystems, which rely on scientific justification and governmental implementation and cannot be passed on to the consumers. It is particularly the decisions according to personal lifestyle, values, and beliefs that ought to be transferred to the consumer, because no institution can competently decide on them in behalf of the individual. To that end an adequate and accurate division between risk and value considerations needs to be performed. In fact, here the question touched to what extent food is and should be a matter of privacy (and self-responsibility)–a topic that has extensive potential for further research and social discussion.

As part of the research consortium “Ethical, legal and socioeconomic aspects of genome editing in agriculture” (ELSA-GEA)^3^, funded by the German Federal Ministry of Education and Research (BMBF), we analyze the requirements a food label has to meet in order to function as an institution for value deliberations. We assume that labels do not only inform consumers about the qualities of available products, but function as a means to communicate preferences by purchasing or rejecting specifically labeled products. Understood that way, food labeling can also serve as an institution or platform for the negotiation of values. To that end, it should, for one thing, not intermingle risk and value aspects. In our view, this stipulation is not answered satisfactorily by current German mandatory and positive GMO labeling practice^4^. Although Kolodinsky and Lusk found that, in the case of Vermont (US), the mandatory label led to an improvement of the public attitude toward genetically engineered food (Kolodinsky and Lusk, 2018), a trust-improving effect (Slovic et al., 1986; Lusk et al., 2014; Kolodinsky, 2018) has not been reported for Germany (Christoph et al., 2008; BMU–Bundesministerium für Umwelt, 2018). Hiding the information about the use of genetic engineering techniques in the fine print and on the backside of product packaging and the exclusion of the majority of those products from the market is likely to contribute to the assumption that–although the product was proven to be safe–there is still something wrong with it. Along with other studies, claiming that a mandatory GMO label increases the apprehensions of consumers (Carter and Gruère, 2003; Zepeda et al., 2003; Sunstein, 2017), we doubt that extending the German GMO label to genome edited products will foster value-based consumer decisions. Secondly, labeling of GE products should not be biased toward a concrete value or value decision. That means, that consumers, who disagree with the use of the technology in agriculture, should be able to identify and purchase products that were not produced with genome editing. However, people who endorse the use of genome editing for specific reasons, should be given information that allows them to actively support the realization of their values. Therefore, the information content of a potential GE label has to exceed the fact of the mere use of the technology in the production process. It should indicate to which end the technique was used and supplementary information concerning practices and challenges in agriculture and food production should be easily available. However, increasing the information content of a label is always associated with the risk of subverting its orientation function due to information overload (Verbeke, 2005; Kronberger et al., 2012). As a third aspect, comprehensibility and clarity have therefore to be taken into account. To a certain degree, new technical solutions such as QR codes^5^could help to mediate between the demands for information supply and clarity. But ultimately, a label that assigns priority to being comprehensive rather than informative also runs the risk of forsaking the orientation function by simplifying too much and encouraging misinterpretations, such as the assumption that GMO labeling is a safety warning. In our opinion such a label does not meet the requirements for mandatory labeling, which should provide absolutely necessary information and therefore must not be ambiguous. In other words, we argue in favor of GE and non-GE labels designed to communicate relevant information for value decisions by the consumer. Reflecting the current situation in Germany, this cannot be achieved by using the existing GMO label. Instead new meaningful GE labels should be designed and issued. To that end producers and governmental institutions have to engage with societal values in the context of agriculture, thereby probably already improving the use of the technology or even the technology itself (Nowotny, 2006).

## Concluding remarks

When new technologies are invented not only do we have to consider whether they are safe to use, but also wherefore and how we want to use said technologies. Therefore, not only scientific knowledge is relevant, but also practical aspects of the application of the technology and societal goals that may be realized or threatened by the technology. However, those situations always confront us with the problem of dealing with divergent but nevertheless legitimate goals within a society. While safety issues regarding the technology itself may be sufficiently examined by scientific means and can be subjected to regulatory policies accordingly, dealing with values requires tolerance, continuing communication and the possibility of coexistence. Consumer communication via labeling offers a good means to govern the desirable variety of legitimate preferences within a society. But only if those divergent preferences are not communicated as mutual threats. The foreseeable necessity to label genome edited products should be seen as an opportunity to institutionalize a comprehensive debate about values relevant in the agricultural context by connecting technological knowledge, societal goals, and individual consumption decisions.

## Author contributions

The author confirms being the sole contributor of this work and has approved it for publication.

### Conflict of interest statement

The author declares that the research was conducted in the absence of any commercial or financial relationships that could be construed as a potential conflict of interest.

## Footnotes

^1^A comprehensive analysis of the strong and the weak interpretation of the precautionary principle was done by Rippe (2001).

^2^For more information visit the TTN website: http://www.ttn-institut.de

^3^For more information visit the ELSA-GEA website: www.dialog-gea.de

^4^For detailed analysis with different types of labels and their effects on markets, view for example: (Gruere and Rao, 2007; Bonroy and Constantatos, 2014).

^5^QR codes are product specific labels which do not provide information immediately, but need to be scanned by electronic devices such as smartphones. When used to provide product information for consumers they usually lead him or her to a website offering detailed information.
